# A collagen membrane for periosteal expansion osteogenesis using a timed-release system in rabbit calvaria

**DOI:** 10.1186/s40729-022-00407-5

**Published:** 2022-03-04

**Authors:** Kensuke Yamauchi, Kazuhiro Imoto, Kenji Odajima, Hiromitsu Morishima, Yoshinaka Shimizu, Shinnosuke Nogami, Tetsu Takahashi

**Affiliations:** 1 Division of Oral and Maxillofacial Surgery, Department of Oral Medicine and Surgery, Graduate School of Dentistry, Tohoku University, 4-1 Seiryo-machi, Aoba-ku, Sendai, Miyagi 980-8575 Japan; 2 Division of Oral Pathology, Department of Oral Medicine and Surgery, Graduate School of Dentistry, Tohoku University, 4-1 Seiryo-machi, Aoba-ku, Sendai, Miyagi 980-8575 Japan

## Abstract

**Background:**

The purpose of this study was to evaluate the effects of resorbable membranes, combined with a shape memory alloy (SMA) mesh device, on bone formation using a timed-release system for periosteal expansion osteogenesis (TIME-PEO).

**Materials and methods:**

Twelve Japanese white rabbits were used in this study. An SMA device was inserted under the forehead periosteum, pushed and bent for attachment to the bone surface, and then fixed using resorbable thread. The rabbits were divided into four groups: C1 (5 weeks postoperatively without membrane), C2 (8 weeks postoperatively without membrane), E1 (5 weeks postoperatively with membrane), and E2 (8 weeks postoperatively with membrane). The rabbits were killed 5 or 8 weeks after the operation and the newly formed bone was assessed histologically and radiographically.

**Results:**

SMA devices, concealed under soft tissue until the time of euthanasia, did not cause active inflammation. The mean activation height, from the original bone surface to the midpoint of the mesh, was 3.1 ± 0.6 mm. Newly formed bone was observed, and most of the subperiosteal space underneath the device was occupied by fibrous tissue. Immature bone was present at the outer surface of the original skull bone in all groups. On histomorphometric analysis, there was no significant difference in the volume of the new bone between C1 and E1 (*p* = 0.885), and C2 and E2 (*p* = 0.545).

**Conclusions:**

PEO using an SMA mesh device, which is based on guided bone regeneration (in atrophic alveolar bone), shows promise as an alternative for bone augmentation, irrespective of whether a resorbable membrane is used.

Augmentation of the alveolar process is a standard procedure during dental implantation because of the lack of alveolar bone height and width. However, the standard autogenous bone grafting technique is associated with donor site morbidity and resorption of grafted bone [1, 2]. Furthermore, it is unsuitable for simultaneous soft tissue augmentation. Distraction osteogenesis (DO) is an alternative method involving the formation of new bone between segments that are gradually separated [3–5]. Furthermore, osteogenesis via periosteal distraction or elevation, without corticotomy, has been suggested as a bone-augmentation technique [6, 7]. This technique facilitates new bone formation without autogenous grafting or osteotomy.

We previously investigated the clinical utility of periosteal expansion osteogenesis (PEO), which was found to be identical to periosteal DO and elevation [8, 9]. We reported the development of a simple, self-activated mesh device composed of NiTi shape memory alloy (SMA) for periosteal expansion, which can create an ideal amount of space via an automated bioactive system combined with an absorbable device [10–12]. The system, including the periosteum, is completely covered by the soft tissue, without penetration of the mucosa or skin. This method is based on the outer dynamic movement of the bone–soft tissue interface, and is referred to as guided bone regeneration (GBR).

In GBR, a mechanical barrier is positioned to prevent rapid fibroblast proliferation, allowing osteoprogenitor cells to repopulate the area to promote bone formation [13, 14]. The use of a resorbable membrane obviates the need for secondary surgery to remove membranes, and reduces the risk of perforation, exposure, and infection [15, 16]. When using titanium mesh, connective tissue formed because of insufficient cell blockade and failure of the peripheral seal at the interface between overlying soft tissue and bone. Experimental and clinical studies have evaluated the combination of titanium mesh and a resorbable membrane for bone augmentation [17, 18]; however, the efficacy of resorbable membranes remains unclear.

We evaluated the effect of a resorbable membrane in combination with an SMA mesh device on bone formation, using a timed-release system for periosteal expansion osteogenesis (TIME-PEO).

## Materials and methods

### Device description

A NiTi mesh device was used (Ni, 56.1 wt%; Ti balance; Fe, 0.05 wt%; O, 0.05 wt%; C, 0.03 wt%; N, 0.02 wt%). The mesh device was 5 mm in width, 25 mm in length, and 0.275 mm in thickness. The mesh was curved such that the middle point was 4 mm above the “baseline”.

The deformation behavior of the SMA mesh plate was assessed by compressive testing. The plate was fixed to an Instron testing machine (Model AG-I; Shimadzu, Kyoto, Japan) and compressive stress was applied perpendicularly at a crosshead speed of 1.0 mm min^−1^. Compressive load and displacement were calculated when the plate had completely flattened.

The center region and entire plate were 5 and 15 mm in width, respectively. The compressive load required for identical displacement of the two regions is 2.7 N (= 0.9 ÷ 5 × 15). This is lower than the measured compressive load applied to the whole plate, likely due to the porosity of the center of the plate.

### Surgical protocol

Male Japanese white rabbits (3–3.5 kg) were divided into four groups of three, according to the time point of euthanasia and collagen membrane coverage status. The protocol and guidelines for this study were reviewed and approved by the Animal Care and Use Committee of Tohoku University, Sendai, Japan (2018SHIDO-030).

The animals were anaesthetized by intramuscular administration of ketamine hydrochloride (60 mg/kg Ketalar; Sankyo, Tokyo, Japan), followed by diazepam (5 mg) and atropine sulphate (0.5 mg), without endotracheal intubation. Before the operation, 10 mg/kg pentobarbital sodium was injected intravenously. In addition, 1.8 mL of local anesthetic (2% xylocaine and epinephrine 1:80,000; Dentsply Sankin, Tokyo, Japan) were used during all surgical procedures.

**Fig. 1:**
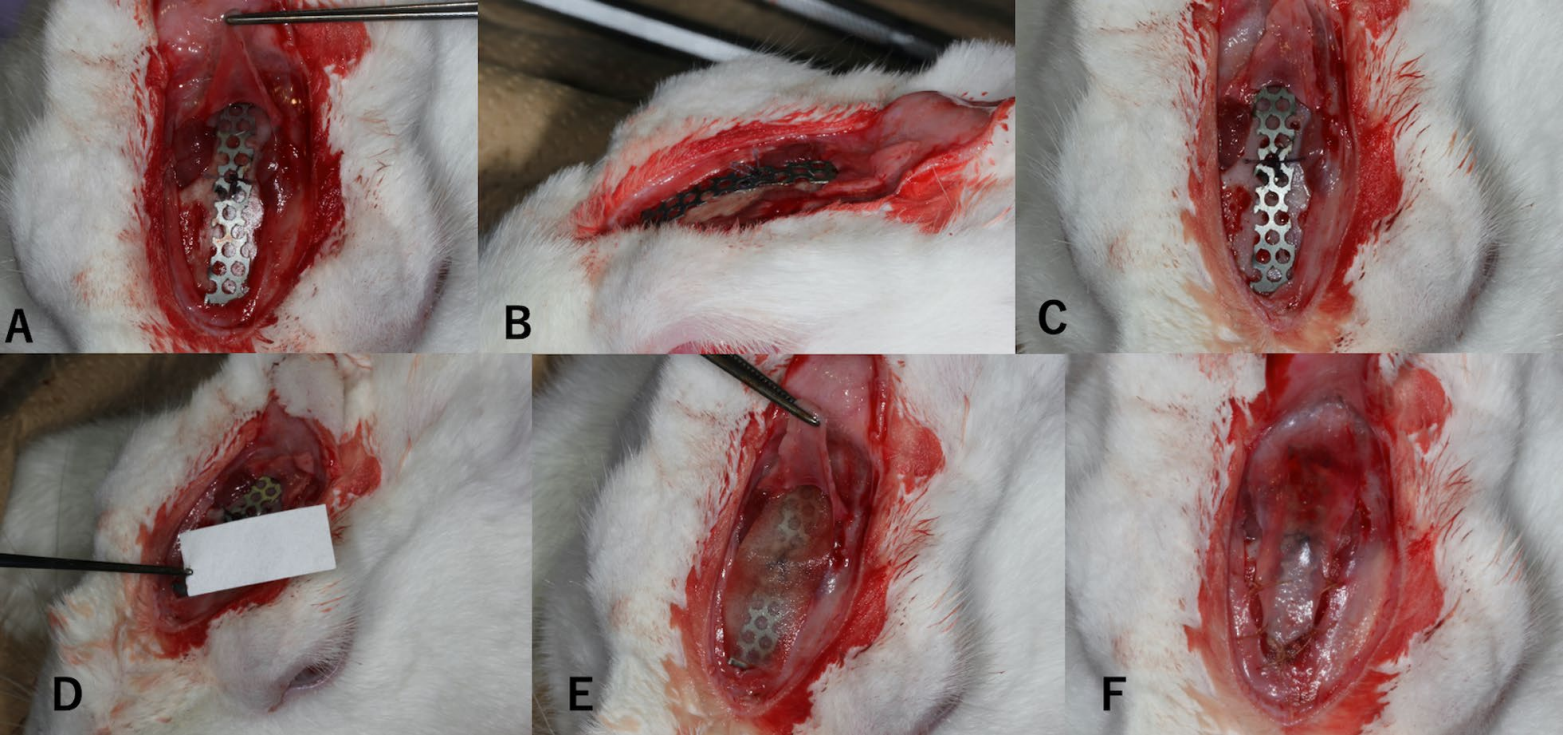
Images of the surgical procedure. **A** and **B** Shape memory alloy (SMA) device fixation using absorbable thread. **C** Appearance after decortication at the original bone surface. **D** Collagen membrane used in the experimental group. **E** Membrane placed over an SMA device. **F** Periosteum closure over the membrane and SMA device

The operation was performed under standard sterile conditions. The forehead of the animal was shaved and disinfected with 1% iodine sodium. After U-shaped skin and periosteal incisions were made in the forehead region, the frontal bone of the animal was exposed following careful elevation of the periosteum. A titanium screw (1.4 mm in diameter, 3 mm in length; Jeil Medical, Seoul, Korea) was inserted into the bone surface to anchor the SMA device using resorbable threads. The device was attached to the bony surface and fixed with 5–0 Vicryl Rapide® resorbable thread (Johnson & Johnson, New Brunswick, NJ, USA). The cortical bone was perforated by drilling through the SMA mesh using a round bur. In half of the rabbits in the experimental group, the SMA device was covered with a resorbable atelocollagen membrane (Koken Tissue Guide; Olympus Terumo Biomaterials, Tokyo, Japan). After irrigation with saline, the periosteum was replaced and stabilized using 5–0 Vicryl (Johnson & Johnson) sutures (Fig. 1). The skin was closed using 4–0 Vicryl sutures. Animals received cefazolin sodium (20 mg/kg) subcutaneously until postoperative day 3.

The rabbits were provided with water and a commercial rabbit diet postoperatively. Rabbits were euthanized with a lethal dose of thiopental sodium at 5 or 8 weeks after surgery. The cranial bone was removed and fixed for 14 days in 10% buffered formalin. The rabbits were divided into groups C1 (5 weeks postoperatively without membrane; *n* = 3), C2 (8 weeks postoperatively without membrane; *n* = 3), E1 (5 weeks postoperatively with membrane; *n* = 3), and E2 (8 weeks postoperatively with membrane; *n* = 3).

### Tissue preparation and histological evaluation

**Fig. 2:**
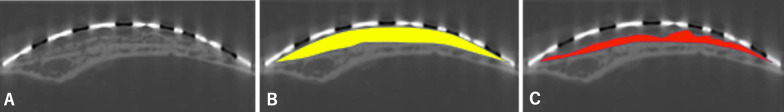
Protocol for histomorphometric analysis using X-ray computed tomography (CT). **A** Original image. **B** The entire area beneath the SMA device was defined as the expanded volume (EV). **C** The area of mineralized tissue in the EV was defined as the total bone volume (TBV)

Cranial bone tissue was evaluated by X-ray computed tomography (CT) (Comscantecno, Co., Ltd., Yokohama, Japan) operating at 65 µA and 70 kV. Measurements were made on three vertical images per specimen, as close as possible to the center of the device. In each image, the area occupied by new bone was measured using ImageJ software (ver. 1.44; NIH, Bethesda, MD, USA). The area beneath the SMA device was defined as the expanded volume (EV). The area of mineralized tissue in the EV was defined as the total bone volume (TBV) (Fig. 2). The EV/TBV ratio was calculated to assess the extent of new bone growth.

After micro-CT, specimens were decalcified in phosphate-buffered saline (PBS) with 10% ethylenediaminetetraacetic acid (EDTA) at room temperature for 60 days. The specimens were then dehydrated in ethanol, cleared in xylene, and embedded in paraffin. Sagittal sections of 5 µm thickness were made using a microtome and mounted on glass slides. Hematoxylin and eosin and elastica and Masson staining were performed on serial sections for morphological evaluation of newly formed bone in the gap between the SMA device and surface of the original cranial bone.

### Statistical analysis

Normality and homogeneity were analyzed first, and an unpaired Student’s *t*-test was used to evaluate the area of newly formed bone and the EV/TBV ratio. The level of significance was set at *P* < 0.05.

## Results

No complications related to the materials used, including infection within or around the device, were observed in any animal at any point during the study period. SMA devices were concealed under soft tissue until the time of euthanasia, and no active inflammation was observed. All devices had returned to their original shape at the time of euthanasia. The mean activation height from the original bone surface to the middle point of the mesh in all rabbits was 3.1 ± 0.6 mm.

### CT evaluation and histomorphometric analysis

**Fig. 3:**
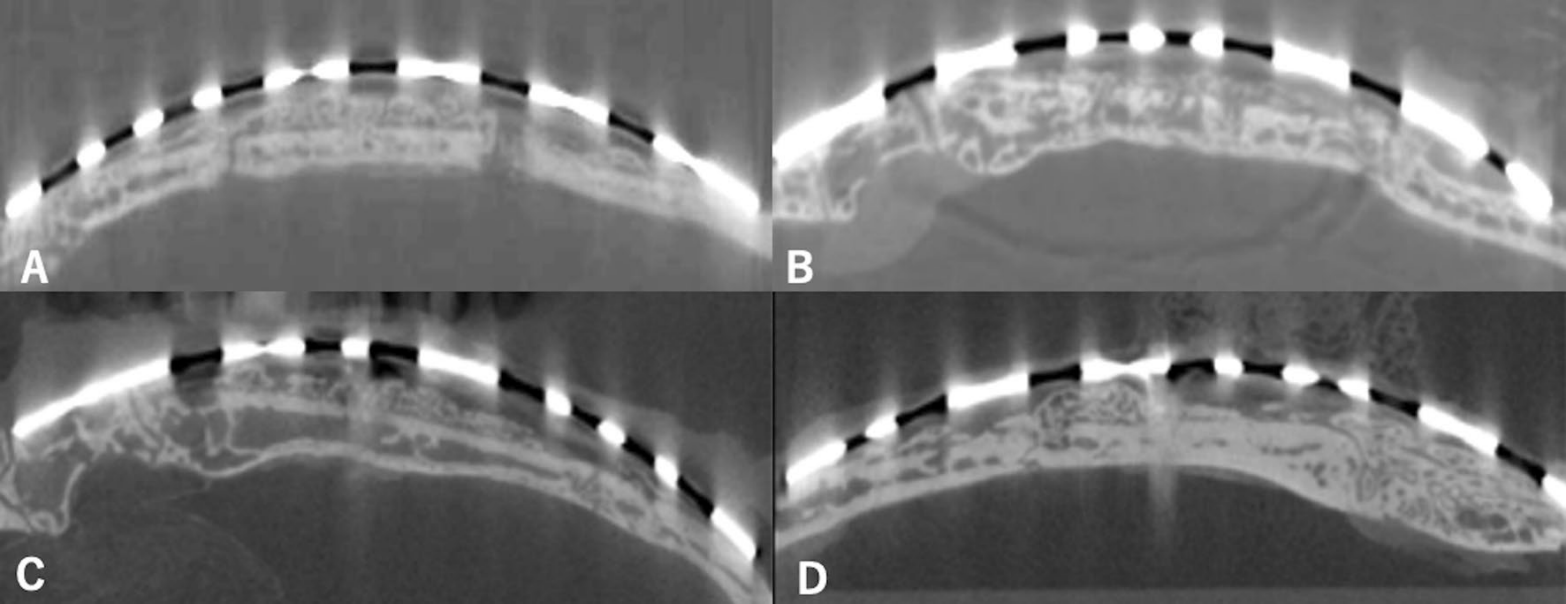
X-ray CT images showing a sagittal cross-sectional view of a dome-shaped bone with the same curvature as the original mesh at 5 weeks postoperatively in C1 (**A**), 8 weeks postoperatively in C2 (**B**), 5 weeks postoperatively in E1 (**C**), and 8 weeks postoperatively in E2 (**D**)

The newly formed bone was less radiopaque than the original bone on X-ray CT images. Newly formed bone was observed, most of the subperiosteal space underneath the device was filled with fibrous tissue, and immature bone was present at the outer surface of the original skull bone in both groups (Fig. 3). The mean new bone volume and TBV/EV ratio are shown in Table 1. According to histomorphometric analysis, there was no significant difference in the volume of new bone between groups C1 and E1 (*P* = 0.885) or C2 and E2 (*P* = 0.545). The TBV/EV ratio data indicated that the membrane did not promote new bone formation during the consolidation period when using the TIME-PEO technique.

**Table 1:** Quantitative data by area and extent of newly formed bone (TBV/EV ratio)

	Post-ope. 5 weeks (mean ± SD)		Post-ope. 8 weeks (mean ± SD)	
	C1	E1	C2	E2
Ratio of TBV/EV (%)	55.8 ± 12.2	54.7 ± 13.1	57.2 ± 11.7	61.4 ± 11.3
*p* value	0.885		0.545	

Group C1 (5 weeks postoperatively without membrane). Group C2 (8 weeks postoperatively without membrane group). Group E1 (5 weeks postoperatively with membrane group). Group E2 (8 weeks postoperatively with membrane group)

### Histological findings

#### Without the membrane

**Fig. 4:**
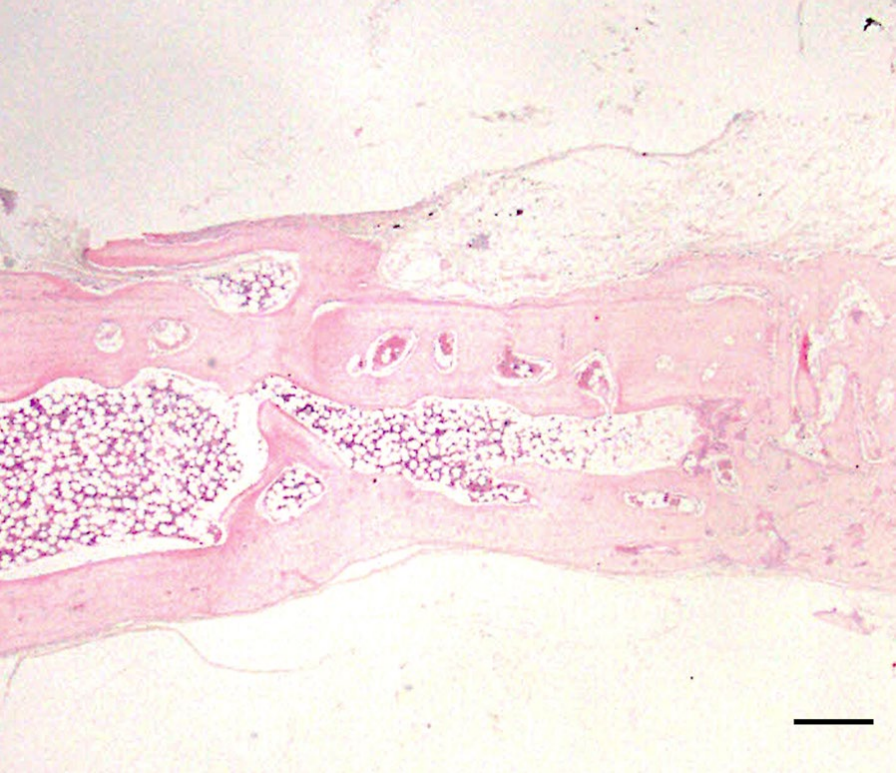
Representative histological hematoxylin and eosin-stained images (C1). An osteogenic layer was observed, which was connected to the original bone by newly formed bony trabeculae at 5 weeks postoperatively. Scale = 1 mm

**Fig. 5:**
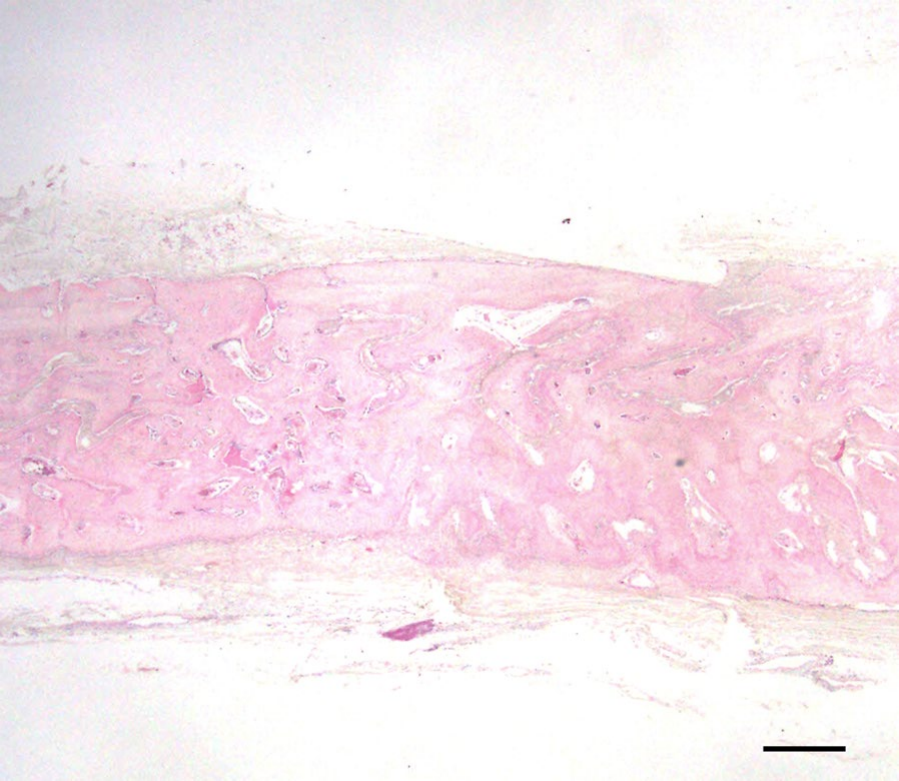
Representative histological hematoxylin and eosin-stained images (C1). Multiple dome-shaped bony trabeculae were scattered over the original bone surface at 8 weeks postoperatively. Scale = 1 mm

**Fig. 6:**
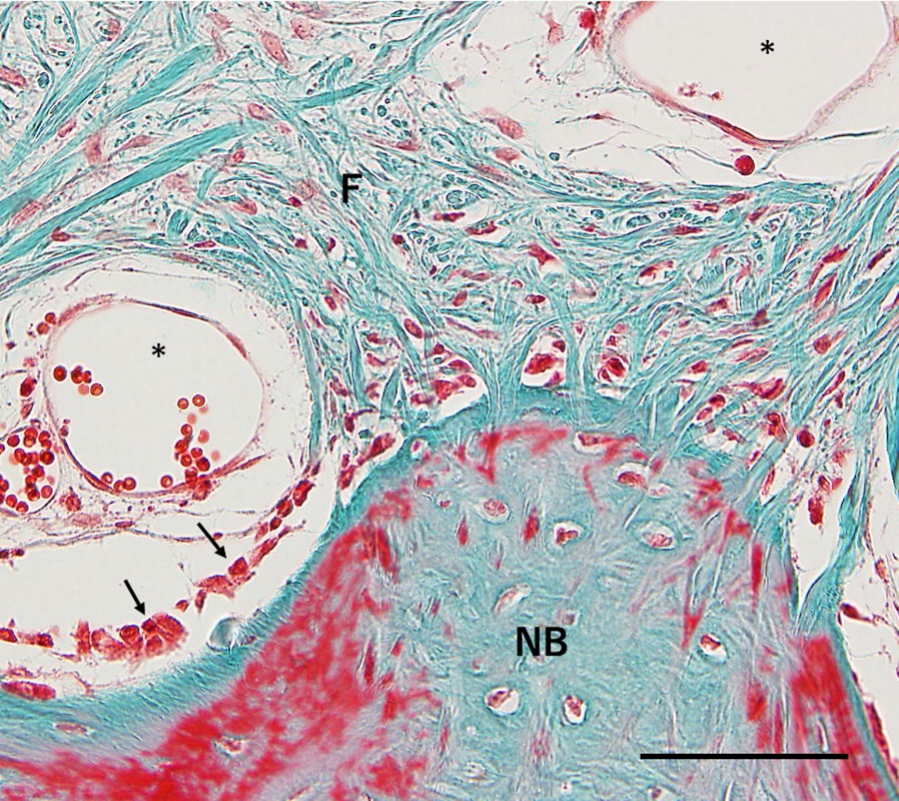
High-power view of the interaction between newly formed bone and the surrounding tissue (C2; Elastica and Masson stain). Fibrous tissue, including sinusoidal vessels, was observed above the newly formed bone. Osteoblasts (black arrowheads) were observed adjacent to the newly formed bone. *Sinusoidal vessel; *F* fibrous tissue, *NB* newly formed bone. Scale = 100 µm

In group C1, most of the subperiosteal space beneath the device was occupied by fibrous tissue, and no inflammatory cells were evident. An osteogenic layer was observed, which was connected to the original bone by newly formed bony trabeculae (Fig. 4). In group C2, multiple dome-shaped, thin bony trabeculae were scattered over the original bone surface, and the interface between new and original bone was unclear compared to group C1 (Fig. 5). Osteoblasts were observed at the interface between fibrous tissue and newly formed bone, and sinusoidal vessels were evident in the surrounding soft tissue (Fig. 6).

#### With the membrane

**Fig. 7:**
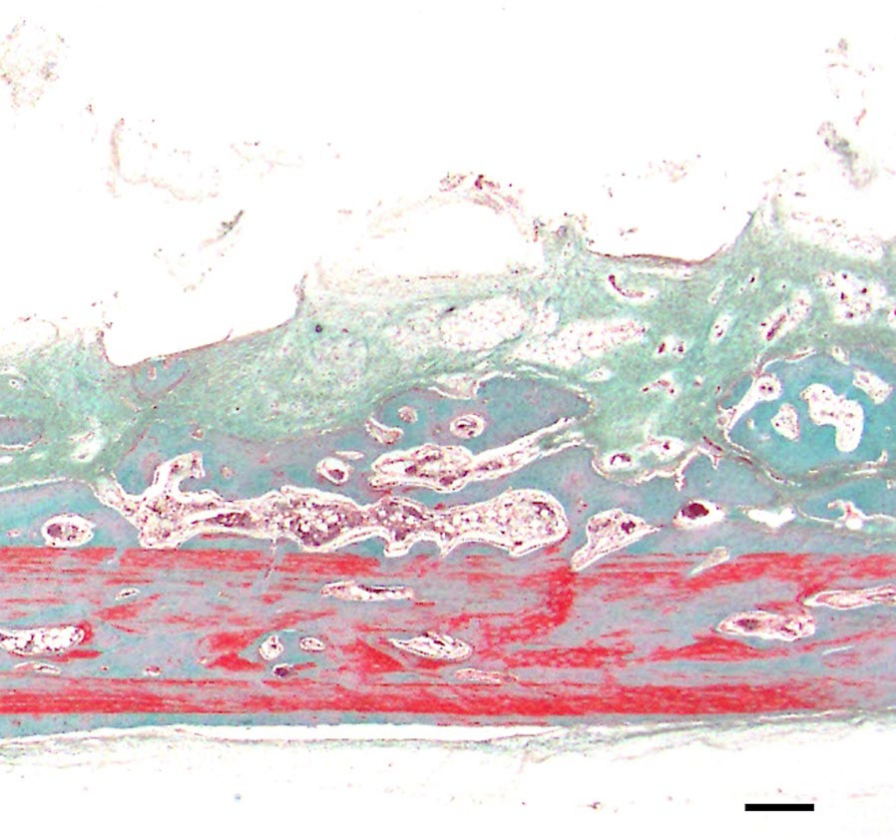
Representative histological Elastica- and Masson-stained images (E1). Multiple newly formed, dome-shaped bony trabeculae were observed over the original bone surface at 5 weeks postoperatively. Scale = 1 mm

**Fig. 8:**
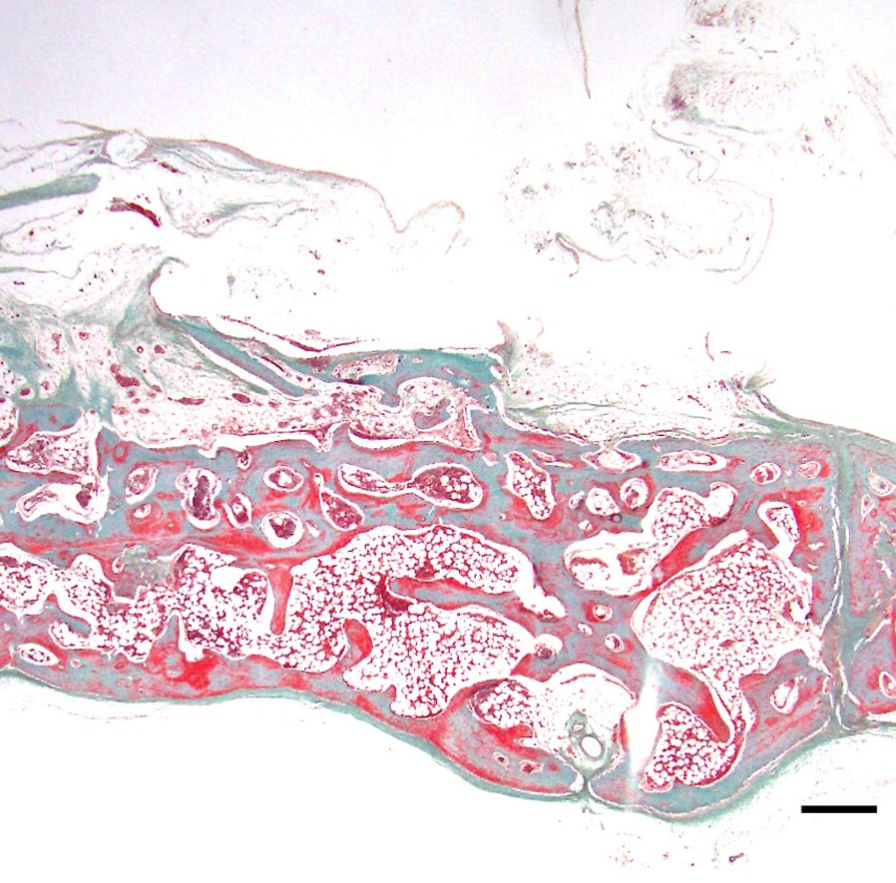
Representative histological Elastica- and Masson-stained images (E2). Multiple dome-shaped bony trabeculae were observed over the original bone surface, and there was dense bone in the cancellous bone area at 8 weeks postoperatively. Scale = 1 mm

**Fig. 9:**
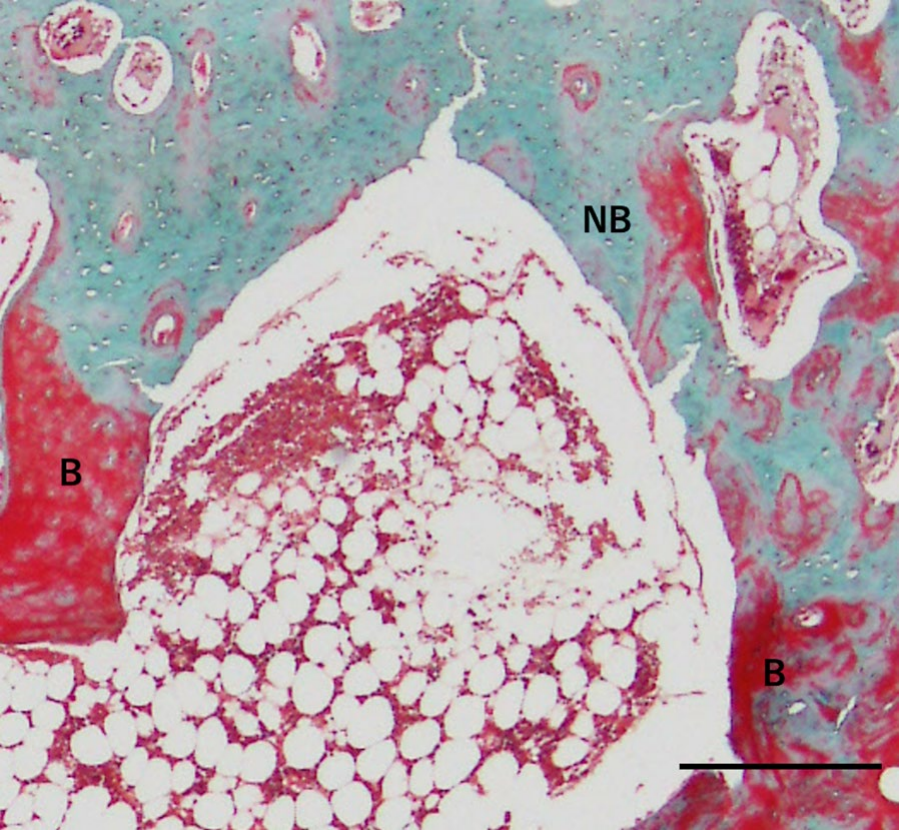
High-power view of the interaction between the newly formed bone and original bone (E1; Elastica and Masson stain). Newly formed bone was observed over the original bone surface around the decorticated area. *NB* newly formed bone, *B* original bone. Scale = 100 µm

**Fig. 10:**
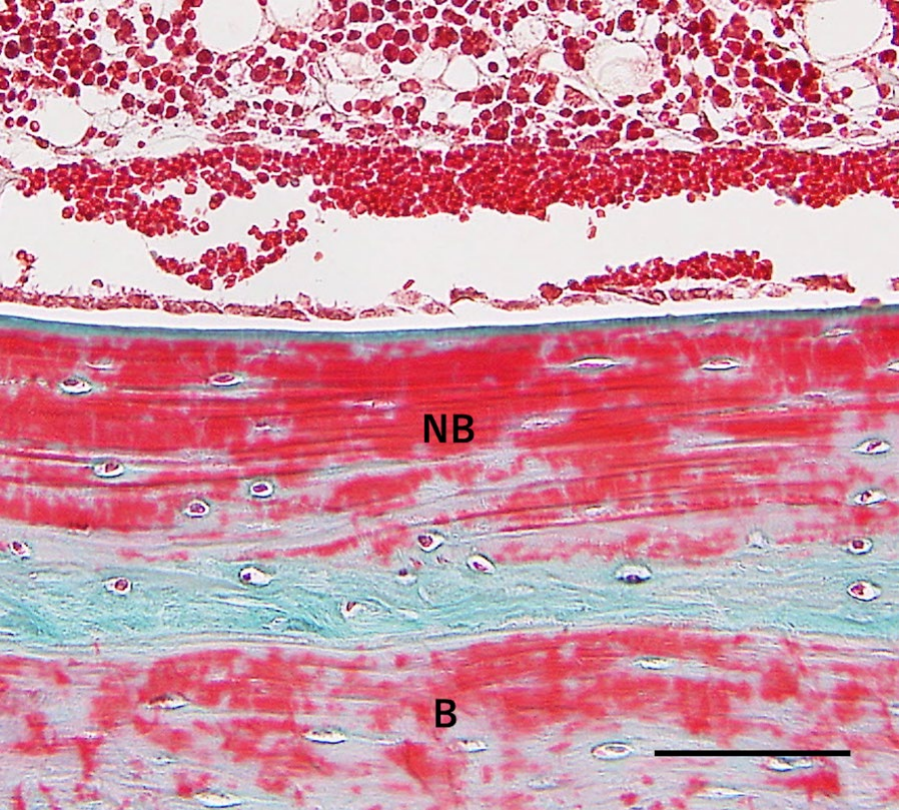
High-power view of the interaction between the newly formed bone and original bone (E2; Elastica and Masson stain). Discoloration and disappearance of the interface between new and original bone. *NB* newly formed bone, *B* original bone. Scale = 100 µm

The subperiosteal space under the mesh device was filled with fibrous tissue, and no bone tissue was observed over the device in group E1 or E2. Multiple newly formed, dome-shaped bony trabeculae were observed over the original bone surface, as in the control group (Figs. 7, 8). Bone formation was also seen in areas adjacent to the cortical perforations; newly formed bone was present over the entire original bone surface (Fig. 9) Elastica and Masson staining revealed maturation of new bone in the E2 group, as evidenced by discoloration and the absence of an interface between the new and original bone (Fig. 10).

## Discussion

In PEO, the SMA device lifts the periosteum via its “shape memory force”, with a contribution from osteoblasts on the periosteal side. This method may be more accurately termed “dynamic GBR”, where there is no requirement for autogenous tissue grafts or artificial bone materials, rather than periosteal distraction or periosteal expansion (which require manual activation) [19]. These results correspond with those of Zakaria et al., who reported that 6–24% of the area directly beneath the titanium mesh device was occupied by new bone after 4 and 6 weeks in a rabbit forehead model [20]. However, Weng et al. reported that new bone occupied 69–77% of the area under a titanium mesh on the outside of monkey mandibles, with or without a barrier membrane, after 4 months [21]. Kostopoulos et al. reported that 52–56% of the space beneath a hemispherical Teflon capsule, on the outside of rat mandibles, was occupied by new bone after 4 months [22]. Also, the newly formed bone occupied 21.9% of the area at 5 weeks postoperatively, and 36.0% at 8 weeks postoperatively [12]. In this study, histomorphometric analysis showed that newly formed bone accounted for 55.8% and 57.2% of all bone at 5 and 8 weeks postoperatively, respectively, in the control group, versus 54.7% and 61.4% in the experimental group. Therefore, the TIME-PEO technique, with or without a membrane, improves augmentation outcomes.

In a GBR procedure, a barrier membrane is placed beneath the periosteum, preventing any effect of osteoblasts in the periosteum [23, 24]. The ideal mechanical barrier for GBR has been studied in terms of occlusivity, stability, pore size, peripheral sealing, bone tissue, the required blood supply, and the ability to promote the proliferation of osteoprogenitor cells [25]. Shin et al. evaluated the effect of a collagen membrane, in conjunction with titanium mesh and an allogenic bone graft, in the context of rabbit calvaria GBR. After 8 weeks, new bone accounted for 10.81 ± 5.38% and 15.16 ± 6.76% of all bone with and without the membrane, respectively; the difference was not significant. Borges et al. evaluated the effectiveness of a collagen membrane combined with titanium meshes for GBR [17]. The collagen membrane was absorbable, being composed of bovine dermal type I atelocollagen. Bone volume and quality were similar with and without the collagen membrane. Nakahara et al. reported that a membrane had no significant impact on the osteogenic response during periosteal DO in rabbit calvaria [26]. By contrast, Saulacic et al. reported that a barrier membrane can promote new bone formation in the context of periosteal distraction [27]. In this study, the collagen membrane did not increase bone formation during PEO.

In this study, epithelial and connective tissue were observed beneath the SMA mesh in both groups, and no bony tissue was observed above the mesh. These findings suggest that fibrous tissue originating from the periosteum penetrated the area in question through the holes in the SMA mesh. Zakaria et al. stated that the speed of the space expansion achieved by periosteal elevation should be optimized, with the ideal rate for optimal bone augmentation being ≤ 0.33 mm/day [28]. This may be because of the lag in the osteogenic response of the periosteum. A higher “periosteal distraction rate” favors growth of the epithelial and connective tissue cells most remote from the original bone surface. Although we detected no significant group differences, the experimental group exhibited a slightly larger newly formed bone area at 5–8 weeks postoperatively compared to the control group. This difference may have been associated with disturbance of epithelial and connective tissue cells in the periosteum by the membranes.

Decortication by perforation of the cortex promotes new bone formation by increasing bleeding, which allows access by progenitor cells and blood vessels, and creates a matrix for “interlocking” of the bone graft with decortified spaces during GBR. Oda et al. were the first to report that cortical bone perforation promoted bony regeneration during periosteal DO, in the mandibular body of a rabbit [29]. Histologically, we found many sinusoidal vessels running from the original cortical perforation into the newly formed bone area. Osteoblasts play a major role in new bone formation; these cells are derived from the periosteum, endosteum, and undifferentiated pluripotent mesenchymal cells of the bone marrow [30]. Vascularization of the original bone marrow promotes early bone regeneration, and sinusoidal vessels might promote vascularization following bone regeneration. Decortication plays an important role in bone regeneration in the gap between the device and residual bone surface.

This study used only one SMA device, and the retention periods and expansion speeds when combining the SMA device with resorbable threads could not be determined. Further large experimental studies are necessary to identify the most appropriate device and the efficacy of resorbable membranes in the alveolar region. The space expansion rate associated with periosteum elevation should be optimized, and the speed thereof should be controlled based on the thickness and curvature of the SMA device, and the condition of the overlying soft tissue.

This study involved rabbits, for which the embryonic origin of the mandible is the same as in humans. The rabbit calvaria is also suitable for bone research, given its simplicity and similarity, in terms of the thick soft tissue layer (including skin) to that of humans [28, 31]. The results of this study suggest that membranes may be unnecessary. However, the outcomes might differ for the human alveola, which has thin, soft tissue with a mucosal layer, and a higher risk of mesh exposure and infection following pure bone formation. Mucosal thickness might be important for wound dehiscence and mesh exposure, and it may be necessary to use membranes in areas with thin biotype mucosa and esthetic zones in the maxillary incisor region.

In our rabbit forehead model, the collagen membrane did not increase bone formation when placed between the periosteum and SMA mesh device. Large animal and human studies are needed to confirm these results.

## Conclusion

PEO using a shape-memory NiTi mesh device shows promise as a clinical alternative for bone augmentation, irrespective of whether a resorbable membrane is used, and applies the concept of dynamic GBR to atrophic alveolar bone.

## Data Availability

The authors of this work are available to support the data.

## Abbreviations

SMA: Shape memory alloy
PEO: Periosteal expansion osteogenesis
DO: Distraction osteogenesis
GBR: Guided bone regeneration
TIME-PEO: Timed-release system for periosteal expansion osteogenesis
EV: Expanded volume
TBV: Total bone volume
